# Corona Poling Enabling Gravure Printing of Electroactive Flexible PVDF-TrFE Devices

**DOI:** 10.3390/ma18010022

**Published:** 2024-12-25

**Authors:** Giuliano Sico, Maria Montanino, Fausta Loffredo, Carmela Borriello, Riccardo Miscioscia

**Affiliations:** Portici Research Centre, ENEA—Italian National Agency for New Technologies, Energy and Sustainable Economic Development, 80055 Portici, Italy; maria.montanino@enea.it (M.M.); fausta.loffredo@enea.it (F.L.); carmela.borriello@enea.it (C.B.); riccardo.miscioscia@enea.it (R.M.)

**Keywords:** energy harvesting, PVDF, PVDF-TrFE, gravure printing, corona poling, piezoelectric, pyroelectric, flexible generator

## Abstract

Polyvinylidene fluoride (PVDF)-based materials are the most researched polymers in the field of energy harvesting. Their production in thin-film form through printing technologies can potentially offer several manufacturing and performance advantages, such as low-cost, low-temperature processing, use of flexible substrates, custom design, low thermal inertia and surface-scaling performance. However, solution-based processes, like printing, miss fine control of the microstructure during film-forming, making it difficult to achieve a high level of polarization, necessary for PVDF to exhibit electroactive characteristics. Here, corona treatment is investigated for the poling of gravure-printed polyvinylidene fluoride–trifluoroethylene (PVDF-TrFE) films, as a particularly suitable poling method for printing since it is rapid, contactless and scalable, and no metal electrodes are required. Effects of corona conditioning on the functional properties of the printed films were examined and discussed. Electroactive properties of corona-poled printed films improved manyfold when they were treated at 9 kV, near room temperature (30 °C) and using very short treatment time (30 s). In particular, piezoelectric and pyroelectric coefficients improved tenfold and by two orders of magnitude, respectively. Considering the upscaling potential of roll-to-roll gravure printing and corona poling, combined with the area-scaling performance of thin-film-based generators, our results can enable the corona-printing process for mass production of future electroactive flexible PVDF-based devices.

## 1. Introduction

Due to the ever-increasing demand for energy and self-powered devices, more and more attention is becoming focused on solid-state harvesting systems based on electroactive materials for converting environmental wasted energy into usable electricity [1,2,3]. To date, the energy scavenged by such converters (range of nW-mW) is proposed to be used for charging low-power or energy storage devices [4,5,6,7,8,9,10,11,12,13,14].

Among the most studied lead-free active materials, polyvinylidene fluoride (PVDF) and its copolymers stand out for the design of energy harvesting systems, thanks to their electroactive and dielectric properties, light weight, flexibility and low-temperature processability [15]. Depending on the processing conditions, semi-crystalline PVDF can crystallize in five different phases. Among them, the polar β phase is recognized for providing the highest electroactive response, originated by a large dipole due to the all-trans configuration of C-F polar covalent bonds [15]. Nonetheless, in order to reach a high macroscopic polarization *Ps* of the polymer, thus obtaining high performance in a PVDF device, β domains must be oriented as much as possible along a preferential direction [11,16]. This alignment can be achieved through a process called poling, which is typically carried out by mechanical stretching at temperatures of 80–140 °C [14,16], by the application of a prolonged electric field in the order of 50–100 MV/m at temperatures in the range of 80–165 °C [17,18] or by processing PVDF by electrospinning [16].

In recent years, conventional printing techniques used in the graphic industry have been widely explored as a novel easy-to-scale-up method for low-cost devices manufacturing in several applications fields, such as electronics [19,20], sensors [21], energy [3,22,23] and emerging biosensors [24,25], internet of things (IoT) [26] and e-textiles [27]. The application of printing technologies can bring several advantages in the production of thin-film PVDF-based devices, including high-throughput, low-cost and low-temperature processing, environmental friendliness, large area, use of flexible substrates and shaping/patterning simultaneously with film deposition [3,28,29]. However, printing typically produces random oriented polycrystalline materials [1,30,31], strongly limiting PVDF performances depending on polarization. For this reason, a poling method specifically suited for printing processes, as opposed to conventionally used methods, has to be implemented in order to manufacture efficient printed devices [18,32].

In this work, for the first time, corona treatment was studied for the poling of gravure-printed polyvinylidene fluoride–trifluoroethylene (PVDF-TrFE) films with the aim to enable printing as a manufacturing method of future flexible, cost-effective and customizable electroactive PVDF-based devices. Corona discharge in the air has the ability to deposit charged ionic species onto the surface of a dielectric layer, generating a secondary electric field across the film, which can potentially be able to align its random crystalline domains [32,33,34]. When corona pin-electrode density and distance are properly set up, uniform surface potential can be established [35]. Accordingly, corona poling appears to be a particularly suitable method to be integrated into the printing process, being an in-air process, contactless, not requiring the deposition of metal electrodes on the film surface and suitable for thin film, large area samples and continuous process [36,37,38]. Corona poling was here tested on dry and wet (still fluid) printed films to find the best compromise between film performance and process parameters.

Among printing techniques, widely industrially used gravure was chosen for this study, thanks to its high scalability and great potential to produce high-quality functional layers at high speed [39,40]. In this regard, the possibility of producing gravure-printed PVDF pyroelectric devices for heat harvesting has been recently demonstrated; however, such unpoled devices showed small pyroelectric currents up to 0.1 nA cm^−2^ at 2.5 K s^−1^ [31].

PVDF-TrFE was selected, since the steric hinderance introduced by TrFE monomer allows the crystallization of PVDF in its high electroactive β phase from a solution, regardless of the process conditions [15,41,42,43]. As a consequence, it is possible to avoid the use of subsequent stretching of the PVDF for inducing α (the most thermodynamically stable, non-polar) to β phase transformation, not compatible with printing film deposition, and to use relatively high temperatures to speed up film drying from printing process solvents (unlike PVDF homopolymer, which cannot exceed 60 °C, causing β phase deterioration [31]).

To study the effects and the effectiveness of the different tested corona conditionings, structural and functional characterizations of the printed films were performed, evaluating their piezo- and pyroelectric responses. Piezoelectricity is the material capability to convert mechanical strain to electrical signal and vice versa, while pyroelectricity refers to the ability of the material to convert temperature change into electrical signal [10]. Such phenomena cause the generation of a current in response to temporal fluctuations, according to the following equations, written in the case of a plane-parallel slab having electrode surfaces perpendicular to the applied stress and a material polar axis under short circuit conditions:

(1) $$i_{piezo}=Ad_{33}\left(d\sigma /dt\right)$$

(2) $$i_{pyro}=Ap\left(dT/dt\right)$$

where A is the surface of the active area of the device (i.e., the area of the electrical contacts), $d_{33}=\varepsilon_{0}E{\left(\partial \varepsilon_{r}⁄\partial \sigma \right)}_{E,T}+{\left(\partial P_{S}/\partial \sigma \right)}_{E,T}$ is the piezoelectric charge coefficient defined as the sum of the changes of relative dielectric constant $\varepsilon_{r}$ and of spontaneous polarization *Ps* with compressive stress σ at constant temperature T and electric field E, $\left(d\sigma /dt\right)$ is the compressive stress σ change with time, $p={\left(dP_{S}/dT\right)}_{\sigma ,E}$ is the primary pyroelectric coefficient of the unclamped material at constant stress *σ* and electric field E and $\left(dT/dt\right)$ is the rate of temperature change [10,32,44]. Since *Ps* is the average electric dipole moment per unit of volume of the material [44], it is clear that the more the polymer dipoles are aligned by poling, the higher the intensity of *Ps* will be, and, consequently, from the above equations, the greater piezo- and pyroelectric response will be achieved [11,45].

## 2. Materials and Methods

Inks suitable for gravure printing were prepared by dissolving PVDF-TrFE 80/20 mol% powder (Mw~200,000, Solvay Specialty Polymers USA LLC, Chicago Heights, IL, USA) under magnetic stirring in a 50/50% wt/wt% of dimethyl sulfoxide (DMSO)/acetone mixture at 60 °C for 1 h.

The inks’ surface tension was determined by a contact angle OCA 20 system (DataPhysics Instruments GmbH, Filderstadt, Germany) in a pendant drop configuration. In order to have good statistics, ten measurements were carried out for each type of ink.

Ink viscosity was measured by a Haake Viscotester iQ (Thermo Fisher, Waltham, MA, USA) at 25 °C.

Inks were gravure printed onto ITO (indium tin oxide)-coated PET (polyethylene terephthalate) film and aluminum foil (both from Sigma Aldrich, St. Louis, MI, USA) using a lab-scale printer G1-5 (IGT, Almere, The Netherlands) with a cylinder having a line density of 40 lines cm^−1^, a stylus angle of 120°, a cell depth of 72 µm and a screen angle of 53°. All the prints were carried out in air. For multilayer printing, each layer was dried at 80 °C for 5 min before printing the next layer to be overlapped.

Corona pre-treatment of the printing substrate was employed for increasing the surface energy of the PET-ITO and to induce partial self-polarization of the PVDF-TrFE via hydrogen bonds [31], using a LabTEC Lab System (Tantec, Lunderskov, Denmark) equipped with a high-frequency generator (range of 25–35 kHz).

The thickness and the surface roughness (*S_q_*) of the prepared samples were examined by a coherence-correlation interferometry-enabled surface profilometer (Taylor-Hobson, model CCI HD4K, Leicester, UK) and by a stylus profiler (KLA, model Tencor P-7, Milpitas, CA, USA).

Prepared samples were also morphologically characterized through scanning electron microscopy (SEM, 1530, LEO Elektronenmikroskopie GmbH, Oberkochen, Germany). The images were acquired with a secondary electron detector on films metallized with a sputtered gold layer.

Structural characterization of the printed films was performed through XRD and Raman spectroscopy analysis. The measurements were carried out on the layers printed on aluminum foil only, due to the superimposition of PVDF signals with ones of the PET substrate. Raman measurements were performed in the wavenumber range of 300–1500 cm^−1^ using a InVia Reflex Raman spectrometer (Renishaw, Torino, Italy) with a laser wavelength of 514.5 nm (laser power 100%) and a 100×-magnification objective. For each investigated sample, several spectra were acquired at different points of the sample surface (by averaging 25 accumulations obtained with 40 s of exposure). The three bands centered at 840 cm^−1^ (symmetric stretching of CF_2_ in trans conformation), at 805 cm^−1^ (stretching of CF_2_ in chains with trans conformation containing gauche defects [46]) and at 1290 cm^−1^ (coupling of CF_2_ stretching and backbone CC stretching and bending modes [46]) were monitored and analyzed. The 840 cm^−1^ band was generally used as a marker for the presence of β crystallites in PVDF and PVDF-TrFE samples [46,47], while the band centered at 805 cm^−1^ was ascribed to the presence of a disordered phase containing gauche defects [46,47]. The third investigated band, centered to 1290 cm^−1^, was also associated in literature with the β form in the PVDF-TrFE, with long trans sequences [46,47,48,49,50]. The XRD results were performed by a X’Pert MDP DY872 X-ray diffractometer (Malvern Panalytical Ltd., Malvern, UK) with Cu-Kα radiation (wavelength 0.154 nm) operating at 40 kV and 40 mA.

Corona poling of the printed samples was carried out using a DC Corona Poling System PK-C30kV-100C (PolyK Technologies LLC, State College, PA, USA) with a fixed distance of 1 cm from the multi-pin electrode to the sample surface to be treated. The pins were arranged in a net where the distance between them is 0.5 cm. Corona poling was tested on dry and wet printed films, changing voltage, duration and temperature as reported in the following section.

Dielectric displacement versus electrical field (D-E) hysteresis loop measurements were carried out at room temperature on some capacitive device samples (PET-ITO/PVDF-TrFe/Cu structure having Cu area of 1 × 1 cm^2^) by a homemade Sawyer–Tower circuit. This, consisting of the series of the device under test (DUT) and an adjustable reference capacitor, was powered by a triangularly shaped signal generated through a EDU33212A arbitrary waveform generator (Keysight Technologies, Santa Rosa, CA, USA) connected in cascade to a TREK 2205-CE solid-state high-voltage amplifier (Trek Inc., Fort Collins, CO, USA)with a fixed gain of 50 V/V. The reference capacitor was made using a capacitor box and the signal across it decoupled from the Y channel of an oscilloscope using an LF412 amplifier (Texas Instruments, Dallas, TX, USA) with a non-inverting buffer circuit configuration. The hysteresis loops were acquired through a benchtop two-channel EDUX1052A oscilloscope (Keysight Technologies, Santa Rosa, CA, USA) working in X-Y mode, representing the reference potential with respect to the input signal to the Sawyer-Tower circuit and acquiring the sampled time series for further processing.

Piezoelectric properties of the printed films were evaluated by directly measuring the piezoelectric constant *d_33_* values using a static force of 2 N and a dynamic force of 0.25 N at a frequency of 110 Hz through a Quasi Static Piezo d_33_-meter PKD3-2000 (PolyK Technologies LLC, State College, PA, USA), equipped with a static force sensor.

Pyroelectric characteristics of the prepared samples were evaluated by imposing controlled temperature ramps on prepared capacitive devices through a HFS600E-PB4 test chamber (Linkam Scientific Instruments Ltd., Salfords, UK) operated through a T96 temperature controller (Linkam Scientific Instruments Ltd., Salfords, UK) equipped with a LNP 96 cooling system (Linkam Scientific Instruments Ltd., Salfords, UK) and recording the thermally stimulated short-circuit current versus time through a B2985A electrometer (Keysight Technologies, Santa Rosa, CA, USA). The measurement of the pyroelectric coefficients was performed by evaluating the maximum stimulated current during repeated heating ramps performed at four different temperature rates. After observing that the pyroelectric current reached a stable value after four or more thermal ramp cycles, the pyroelectric coefficient “*p*” was then extracted as the slope of the pyroelectric current versus the thermal rate data for a given sample.

Finally, pyroelectric peak power measurements were performed by simultaneously measuring the pyroelectric current and the voltage at the ends of a self-built resistor box connected to the DUT while applying thermal ramp cycles inside to the Linkam stage. The voltage across the resistor box was recorded by a DMM6500 6½-Digit Benchtop Digital Multimeter (Keithley Instruments, Cleveland, OH, USA). The electrical power data were evaluated as the peaks in instantaneous power on the resistor box for different values of the electric load resistors during the upwards thermal ramps.

## 3. Results and Discussion

### 3.1. Gravure-Printed Film Preparation and Characterization

**Figure 1:**
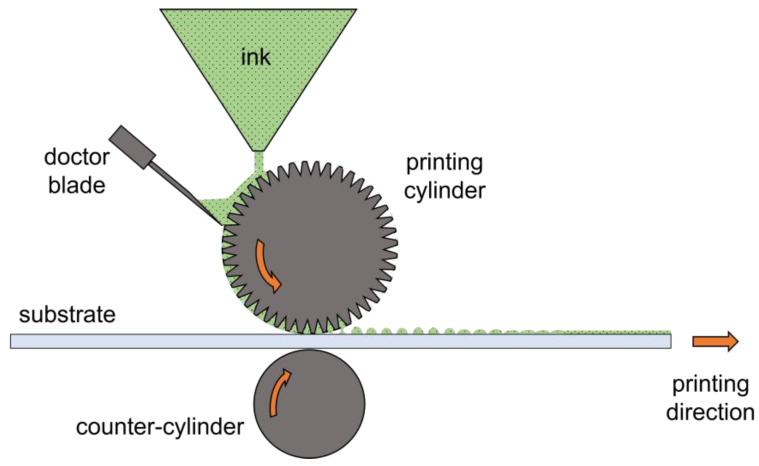
Schematic of roll-based gravure printing operation principle. Adapted with permission from ref. [39].

Gravure printing consists of low-viscosity ink direct transfer from separate micro-engraved cells of a chromed printing cylinder onto a flexible substrate by the pressure of a counter rubber cylinder [51], as depicted in Figure 1. Accordingly, to be properly deposited by gravure, an active material must be applied by means of a multicomponent fluid system (ink), typically having a viscosity below 100 mPa s and a surface tension below 42 mN/m [39].

Therefore, using a DMSO/acetone mixture as the solvent, some inks were prepared by varying PVDF-TrFE concentration, characterizing them from the rheological point of view. As reported in Figure S1, the viscosity trends were independent of the shear rate (Newtonian behavior), and the values were < 100 mPa s for polymer concentrations < 15 wt%. The surface tension of thus prepared inks resulted in being suitable for gravure printing, thanks to the high solvent content having low surface tension value (γ_DMSO_ = 44 mN m^−1^ and γ_acetone_ = 25 mN m^−1^ at 20 °C) (see Figure S2).

Using such inks, printing tests were carried out by varying both printing force (from 300 to 700 N) and speed (from 12 to 60 m min^−1^) in search of the best macroscopic printing quality. To this aim, especially in the case of Newtonian ink, a practical criterion is to adjust the ink and process parameters to have Ca = Uη/γ ≈ 1, where Ca is a dimensionless capillary number, U is the printing speed and η and γ are the viscosity and the surface tension of the ink, respectively [39]. Here, considering the high molecular weight of the polymer, a speed of 12 m min^−1^ was preferred in order to slightly favor the ink spreading (i.e., Ca < 1), so as to limit the possible pinhole development during film-forming. Moreover, multilayer printing was also performed to further avoid the occurrence of possible micro-short circuits in the device, overlapping up to five layers at the same printing conditions (speed of 12 m min^−1^ and printing force of 500 N). Inks were superimposed with decreasing concentration (from 12 to 8 wt%) as the number of layers increased to minimize the stack surface roughness [52]. The morphological characterization of such a multilayer revealed a final film thickness of 3.8 ± 0.3 µm with a *S_q_* of 0.30 ± 0.03 µm.

**Figure 2:**
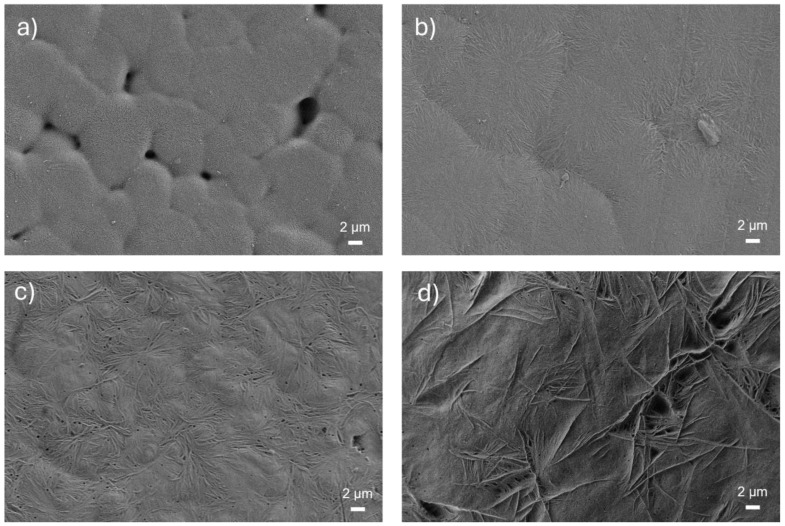
SEM image of the top surface of gravure-printed PVDF-TrFE film thermally annealed for 1 h at different temperatures: (**a**) 100 °C; (**b**) 120 °C; (**c**) 140 °C; (**d**) 160 °C.

**Figure 3:**
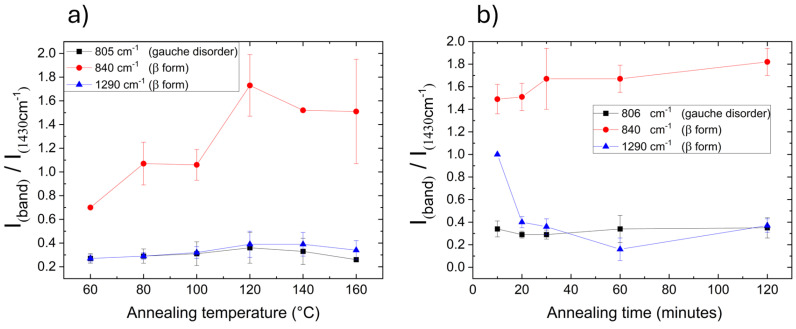
Intensities of different polymer Raman bands (normalized to the band centered at 1430 cm^−1^) measured for 80/20 mol% PVDF-TrFE based films obtained by changing (**a**) the annealing temperature (duration of 1 h) and (**b**) the annealing time (for samples annealed at 120 °C). Error bars were determined from the standard deviation obtained by 10 measurements on different areas of the sample.

As expected, such a PVDF-TrFE multilayer film was composed of only the β polar phase, meeting structural requirement for exhibiting electroactive features (see Figure S3). However, in order to increase the crystalline degree of the film and consequently its potential electroactive response, printed samples were subjected to thermal annealing treatments (from 80 to 160 °C for 1 h) [38,41,53]. At the lowest treatment temperature (80 °C), the film appeared transparent, resulting in being mostly amorphous. As the temperature increased, granular crystallites formed and grew, until their morphology changed to fibrillar-like, as reported in Figure 2. The β phase increased with the annealing temperature, showing a maximum at 120 °C, as showed in Figure 3a. In order to reduce the process time, films were then annealed at 120 °C, changing the treatment time, and it was observed that already after 30 min, the β phase reached the same amount as in 1 h (see Figure 3b).

### 3.2. Corona Poling and Functional Characterization of Gravure-Printed Film

**Figure 4:**
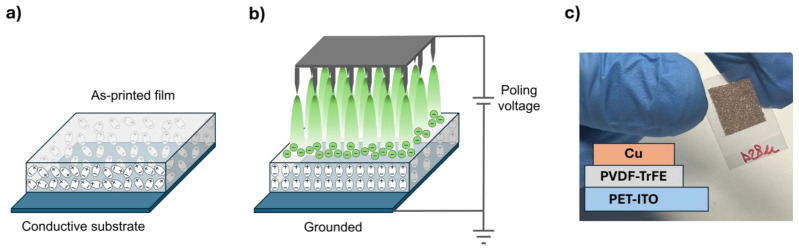
Diagram of the production of corona-poled gravure-printed PVDF-TrFE device: (**a**) as-printed film; (**b**) schematic diagram of corona poling process; (**c**) capacitive device sample for functional characterization.

**Figure 5:**
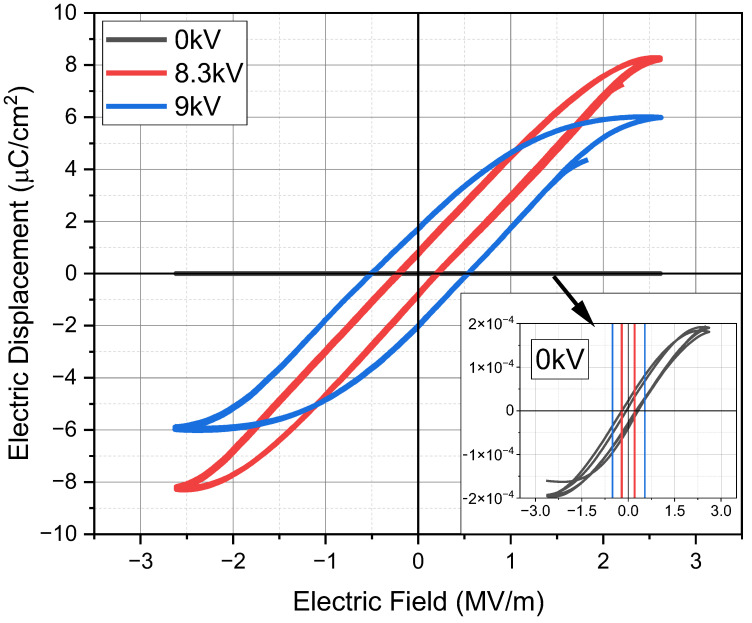
Dielectric displacement versus electrical field hysteresis loop measured for PVDF-TrFE gravure-printed devices as the poling voltage varies. In the inset, the hysteresis loop of the unpoled PVDF-TrFE gravure printed device is shown.

The 30 min annealed printed films were corona poled as the voltage increased (up to 9.5 kV), at a fixed treatment time of 1 min and 30 °C, as schematized in Figure 4a,b. The temperature was chosen to have a stable reference temperature very close to the ambient temperature. To evaluate the effects on film polarization due to corona, ferroelectric, piezoelectric and pyroelectric measurements were carried out. To this aim, capacitive device samples having an area of 1 cm × 1 cm were prepared by spraying a Cu-based varnish for making the upper contact on the printed films (Figure 4c). In Figure 5, D-E loops of some representative capacitor-like treated films as the corona voltage increased are reported. As can be seen, treated samples showed hysteresis loops much larger than the unpoled sample, revealing an abundant presence of ferroelectric domains; moreover, the intercept with the y-axis (net remnant polarization moment) got larger as the poling voltage increased.

The functional characterization involved the measurements of piezo- and pyroelectric characteristics of poled samples. In Table 1, the trend of the piezoelectric *d_33_* coefficient versus the corona voltage is reported. As can be observed, once a threshold voltage was exceeded (>7.5 kV), *d_33_* rapidly increased until reaching a maximum at 9 kV, beyond which electrical arcing occurred, causing dielectric breakdown of the treated samples, as also shown by SEM images (see Figure S4). Such an enhancement was due both to a further increase in β domains (see Figure S5) and to their alignment with the voltage [32,33,38,54]. In addition, crystalline morphology of the films annealed at 120 °C was found to be changed into being fibrillar-like with corona poling at high voltage (see Figure S6). No further improvements were observed when repeating the same treatment for multiple cycles.

**Figure 6:**
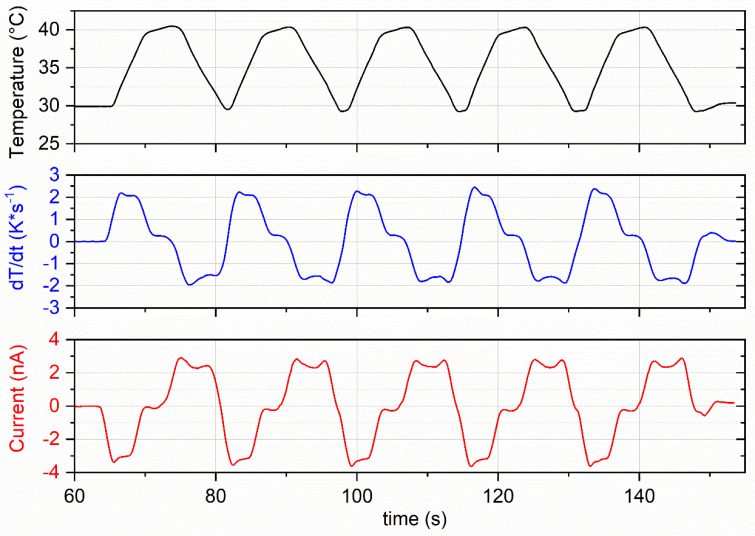
Example of temperature and electric current measured vs. time for a corona-poled gravure-printed PVDF-TrFE device.

The same trend was observed by pyroelectric characterization, imposing controlled thermal ramps at different nominal rates (0.8, 1.7, 2.1 and 2.5 K s^-1^) between 30 and 40 °C setpoints and measuring the short-circuit current. In Table 2, the measured current and pyroelectric coefficient as obtained by Equation (2) are reported, while in Figure 6, an example of data acquisition is presented. As can be seen, the obtained trends are consistent with pyroelectric phenomena, since the measured electric current was proportional to the *dT/dt* shape and the modulus of the pyroelectric current increased proportionally to the temperature rate. Moreover, the pyroelectric characteristics increased as the corona voltage increased, confirming that more and more polymer dipoles can rotate and vertically align in the direction of the electric field generated by the corona voltage growth.

**Figure 7:**
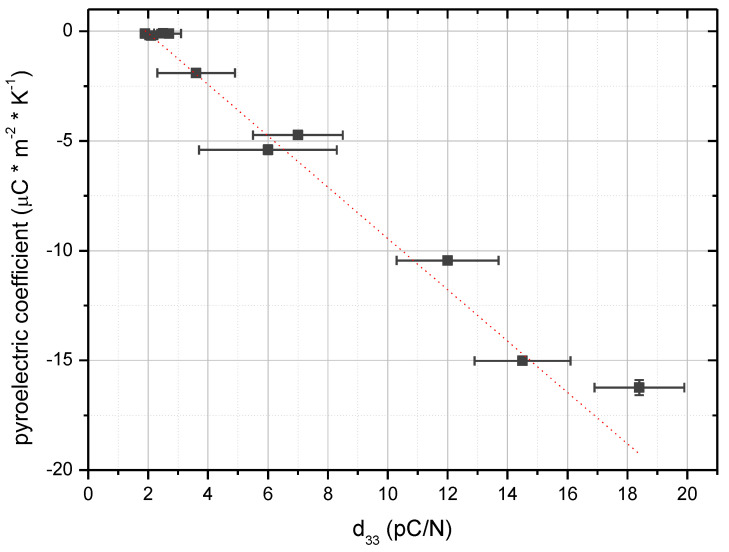
Pyroelectric coefficient (*p*) versus piezoelectric charge coefficient (*d_33_*) for corona-poled PVDF-TrFE gravure-printed devices as the voltage increases at a fixed treatment time of 1 min and 30 °C. Pyroelectric coefficients were estimated by a zero-crossing linear fit of current densities versus temperature rates; error bars referred to the fitting error. d_33_ were determined by averaging 10 measurements; the error bars were determined from the standard deviation of data.

The piezoelectric and pyroelectric trends agreement of the treated samples was expected, since such properties are both proportional to the spontaneous polarization *P_S_*. This is here experimentally evident, where the piezoelectric coefficient is almost linearly proportional to the pyroelectric coefficient, as shown in Figure 7.

In order to study the effect of treatment time, printed films were then corona poled at fixed 9 kV and 30 °C for different times. The results were collected and are displayed in Table 3. After just 30 s, the same properties were obtained as after 1 min and then improved after 2 min, further showing that the *d_33_* and *p* coefficients of the untreated film increased by one and two orders of magnitude, respectively, using corona. Beyond 2 min, electrical discharges became increasingly frequent, damaging the samples.

The corona treatment temperature had no beneficial effect on the electroactive properties of the printed films, which actually worsened as the temperature increased (from 30 to 100 °C), probably due to partial depolarization [32]. On the other hand, in conventional poling processes, high temperatures are typically used to promote molecular mobility under strong electric field and then lowered at the end of the treatment, with the electric field still active to force the domains into the aligned configuration. Nevertheless, a very fast reduction from high to room temperature was not feasible in our case considering the very short treatment times used.

Comparisons of the obtained results with earlier reports are rather limited, since the literature on corona poling, especially applied to PVDF polymers, is still poor, and no examples of other gravure-printed PVDF-based devices have been reported to date. As a result, the best piezo- and pyroelectric characteristics obtained through corona poling of gravure-printed PVDF-TrFE film were in line with the literature tested under similar conditions, showing the same order of magnitude [11,18,43,53]. However, of note, such results were obtained using time and/or temperature and/or voltage lower than those used in other types of corona poling, as summarized in Table 4, and especially in the most used conventional electrical poling [42,43,55,56], demonstrating that corona is particularly suitable for integration into rapid and low-temperature gravure printing processes. Moreover, corona appears particularly effective and uniform when applied to homogeneous and high-quality films like those here produced by gravure printing. Therefore, considering the high upscaling potential of both roll-to-roll gravure printing and corona poling, combined with the area-scaling performance of thin-film-based electroactive generators, our obtained results can enable the corona-printing process for the potential mass production of future flexible electroactive PVDF-based devices.

**Figure 8:**
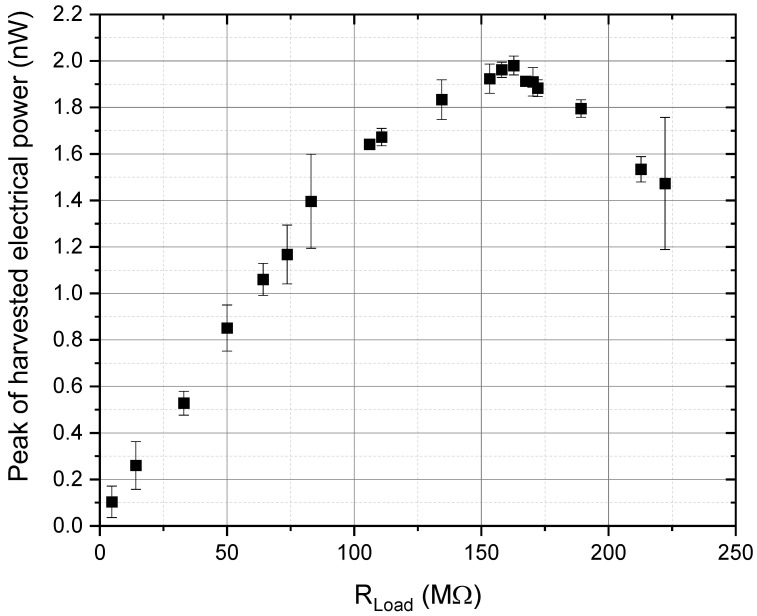
Diagram of the maximum instantaneous harvested electrical power vs. load for gravure-printed PVDF-TrFE poled at 9 kV for 2 min subjected to a thermal variation of 2.5 K s^-1^. Power data were estimated from a population of four samples of current and voltage per resistance value.

To test the energy harvesting performance of poled PVDF-TrFE printed film, the evaluation of the pyroelectric peak power of the best prepared device was also carried out, reaching a maximum peak instantaneous power of 2 nW under an external load of 163 MΩ, as shown in Figure 8; the maximum power transfer is achieved when the external load equals the internal resistance of the generator.

Finally, in order to individuate the best printing-poling processes integration, wet poling on as-printed fluid samples was also tried, taking inspiration from Tansel’s work [59]. In particular, a corona voltage from 5 to 9 kV was tested during isotherms from 50 to 100 °C for 10 to 30 min (so as to match the pre-poling annealing time in the previous dry corona case), then cooling to room temperature under voltage. However, in all tested cases, the piezo- and pyroelectric properties were poor, reaching in the best case (9 kV, 100 °C isotherm for 30 min) 2.7 pC N^−1^ and −9.7 × 10^−1^ nC m^−2^ K^−1^ of piezo- and pyroelectric coefficients, respectively. Structural and morphological analyses were carried out in order to investigate the low performance of wet-poled films. The comparison between the Raman spectra of the best wet-poling sample and the thermally annealed one showed a similar amount of β crystalline form but differences in terms of the disordered form containing gauche defects that was higher for the wet poled sample (see Figure S5). Moreover, the SEM images showed the development of microporosity during film-forming under wet poling (see Figure S7). This was probably the main factor responsible for the low performance. As expected, post-poling thermal annealing further degraded the properties of the wet poled samples due to the depolarization effect by temperature. Therefore, the wet procedure did not appear to be suitable for the gravure printing process at this stage, unless dedicated in-depth future work is carried out.

In summary, corona was successfully used for poling gravure-printed PVDF-TrFE films, dramatically improving their electroactive properties; this is considered to be a particularly suited poling method for coupling with printing. In fact, many advantages are offered by corona over other poling techniques, such as the suitability for thin or low-density films, the short duration, the use of room temperature, the scalability, the absence of contact and metal electrodes and the lack of need for a controlled environment. Such conditions are very attractive for the industry, especially for printing. Therefore, our results can enable the mass production of electroactive flexible PVDF-based devices through simple, low-cost and high-throughput methods, such as printing and corona, promoting sustainable manufacturing practices. Nevertheless, a preliminary study on a suitable voltage avoiding film damage has to be carried out, and the effectiveness of the substrate grounding over a large area in continuous industrial processes has to be considered.

In perspective, the opportunity to manufacture low-cost generators through printing processes having high device customizability, high reproducibility and uniformity, also over a large area, can promote wide use of such devices for energy harvesting in several fields, opening new product opportunities. Among these, medical applications [61] and powering small IoT nodes [12] and wearable and portable devices [1,45] appear to be the closest applications. Moreover, in-line integration of printed generators with emerging printed electronics or with other types of energy generators can be considered as further improvements [3,45].

## 4. Conclusions

In this work, corona was used for successfully poling gravure-printed PVDF-TrFE films. Ferroelectric, piezoelectric and pyroelectric properties of the treated samples improved as increasing the poling voltage in a very short time (30 s) and at near room temperature (30 °C). In particular, at 9 kV, piezoelectric and pyroelectric coefficients improved tenfold and by two orders of magnitude, respectively, reaching literature values obtained using more demanding conditionings and production techniques other than corona and gravure printing. In fact, such a combination offers many advantages: corona poling is a fast, contactless, scalable and room-temperature in-air process, not requiring metal electrodes, while gravure printing is the fastest and highest quality large-area in-air industrial film deposition technique. As a result, corona was demonstrated to be a very efficient method for poling printed PVDF films, enabling a printing-corona combination for future mass production of electroactive flexible devices. Considering the high sustainability and upscale potential of the investigated production processes, the obtained results appear to be very meaningful from a technological point of view. To date, since no examples of gravure-printed PVDF-based devices have been reported, there is an opportunity for additional process and performance optimization, for example, investigating in depth the wet procedure, the effects on temperature change of light-induced phenomena, the introduction of active fillers and how to further decrease the process times and increase the film thickness.

## Figures and Tables

**Table 1:** Values of piezoelectric charge coefficient (*d_33_*) measured for PVDF-TrFE gravure-printed devices vs. corona poling voltage applied at 30 °C.

Poling Voltage (kV)	*d_33_* (pC N^−1^)
0	1.9 ± 0.1
7.5	2.1 ± 0.2
7.8	3.6 ± 1.3
8.0	6.0 ± 2.3
8.3	7.0 ± 1.5
8.6	12.0 ± 1.7
8.8	14.5 ± 1.6
9.0	18.4 ± 1.5

**Table 2:** Values of pyroelectric currents (*i_p_*) measured for different nominal rates *dT/dt* on PVDF-TrFE gravure-printed devices as the poling voltage at a fixed treatment time of 1 min and 30 °C; the average of the pyroelectric coefficient (*p*) is also reported. Raw currents (*i_p_*) data were acquired with an accuracy of 0.2% + 5 pA.

Poling Voltage (kV)	Nominal Rate *dT/dt* (K s^−1^)	*i_p_* (nA)	*p* (µC m^−2^ K^−1^)
0	0.8	−0.9 × 10^−2^	−0.11 ± 0.01
	1.7	−1.8 × 10^−2^	
	2.1	−2.2 × 10^−2^	
	2.5	−2.4 × 10^−2^	
7.5	0.8	−1.8 × 10^−2^	−0.19 ± 0.04
	1.7	−3.2 × 10^−2^	
	2.1	−3.9 × 10^−2^	
	2.5	−4.6 × 10^−2^	
7.8	0.8	−1.6 × 10^−1^	−1.90 ± 0.01
	1.7	−3.2 × 10^−1^	
	2.1	−4.0 × 10^−1^	
	2.5	−4.7 × 10^−1^	
8.0	0.8	−4.2 × 10^−1^	−4.73 ± 0.04
	1.7	−7.9 × 10^−1^	
	2.1	−9.8 × 10^−1^	
	2.5	−1.8	
8.3	0.8	−5.8 × 10^−1^	−5.40 ± 0.21
	1.7	−9.0 × 10^−1^	
	2.1	−1.1	
	2.5	−1.3	
8.6	0.8	−9.5 × 10^−1^	−10.44 ± 0.14
	1.7	−1.8	
	2.1	−2.2	
	2.5	−2.6	
8.8	0.8	−1.3	−15.03 ± 0.12
	1.7	−2.5	
	2.1	−3.1	
	2.5	−3.7	
9.0	0.8	−1.5	−16.24 ± 0.34
	1.7	−2.8	
	2.1	−3.4	
	2.5	−3.9	

**Table 3:** Values of pyroelectric currents (*i_p_*) measured for different nominal rates on PVDF-TrFE gravure-printed devices as corona poling time; the averages of the pyroelectric coefficient (*p*) and piezoelectric coefficient (*d_33_*) are also reported. Pyroelectric currents (*i_p_*) were measured with an accuracy of 0.2% + 5 pA.

Poling Time (min)	Nominal Rate *dT/dt* (K s^−1^)	*i_p_* (nA)	*p* (µC m^−2^ K^−1^)	*d_33_* (pC N^−1^)
0.5	0.8	−1.4	−16.4 ± 0.3	18.2 ± 1.1
	1.7	−2.7		
	2.1	−3.4		
	2.5	−4.1		
1	0.8	−1.5	−16.2 ± 0.3	18.4 ± 1.5
	1.7	−2.8		
	2.1	−3.4		
	2.5	−3.9		
2	0.8	−1.9	−20.7 ± 0.4	19.0 ± 1.0
	1.7	−3.5		
	2.1	−4.3		
	2.5	−5.1		

**Table 4:** Comparison of corona poling used for PVDF-TrFE films; RT = room temperature.

Ref.	Voltage (kV)	Time (min)	Temperature (°C)
This work	9	0.5	30
[57]	7.5	5	RT
[54]	2	30	RT
[34]	26	60	RT
[38]	35	2	RT
[37]	10.5	90	60
[32]	18	30 + 20	110 + RT
[58]	17.5	120 + 60	120 + RT
[59]	20	180	RT
[60]	10	30	125

## Data Availability

The original contributions presented in this study are included in the article/Supplementary Materials. Further inquiries can be directed to the corresponding author.

## Supplementary Materials

The following supporting information can be downloaded at https://www.mdpi.com/article/10.3390/ma18010022/s1: Figure S1: Viscosity vs. shear rate for the inks having 8–15 wt% of PVDF-TrFE 80/20 mol% at 25 °C; Figure S2: Surface tension measurements of PVDF-TrFE 80/20%mol solutions in DMSO/acetone; Figure S3: Raman (a) and XRD (b) spectra of a multilayer PVDF-TrFE 80/20 mol% film deposited by gravure printing and post-annealed at 120 °C for 1 h; Figure S4: Top surface of gravure-printed PVDF-TrFE film corona poled at 9.5 kV, 30 °C for 1 min; Figure S5: Comparison of crystalline phases by Raman spectroscopy of gravure-printed PVDF-TrFE samples: as-printed, thermal annealed at 120 °C for 1 h, corona poled (at 9 kV, 30 °C, 1 min) and wet poled (at 9 kV, 100 °C, 30 min); Figure S6: Top surface of gravure-printed PVDF-TrFE film corona poled (at 9 kV, 30 °C for 1 min) at different magnification: (a) 1 kX; (b) 5 kX; Figure S7: Top surface of wet poled (at 9 kV, 100 °C isotherm for 30 min) gravure-printed PVDF-TrFE films at different magnifications: (a) 1 kX; (b) 5 kX.
