# Supplementary material for: Corona Poling Enabling Gravure Printing of Electroactive Flexible PVDF-TrFE Devices
Source: Materials (Basel). 2024 Dec 25;18(1):22. doi: 10.3390/ma18010022 (PMC11721702; doi:10.3390/ma18010022)
Supplement: Supplementary file 1 [file materials-18-00022-s001.zip › materials-3346115-supplementary.pdf]

materials

MDPI

Article

Supplementary materials: Corona poling enabling gravure printing of electroactive flexible PVDF-TrFE devices

Giuliano Sico *, Maria Montanino, Fausta Loffredo, Carmela Borriello and Riccardo Miscioscia

ENEA - Portici Research Centre, Italian National Agency for New Technologies, Energy and Sustainable Economic Development, 80055 Portici, Italy; maria.montanino@enea.it (M.M.); fausta.loffredo@enea.it (F.L.); carmela.borriello@enea.it (C.B.); riccardo.miscioscia@enea.it (R.M.)

* Correspondence: giuliano.sico@enea.it

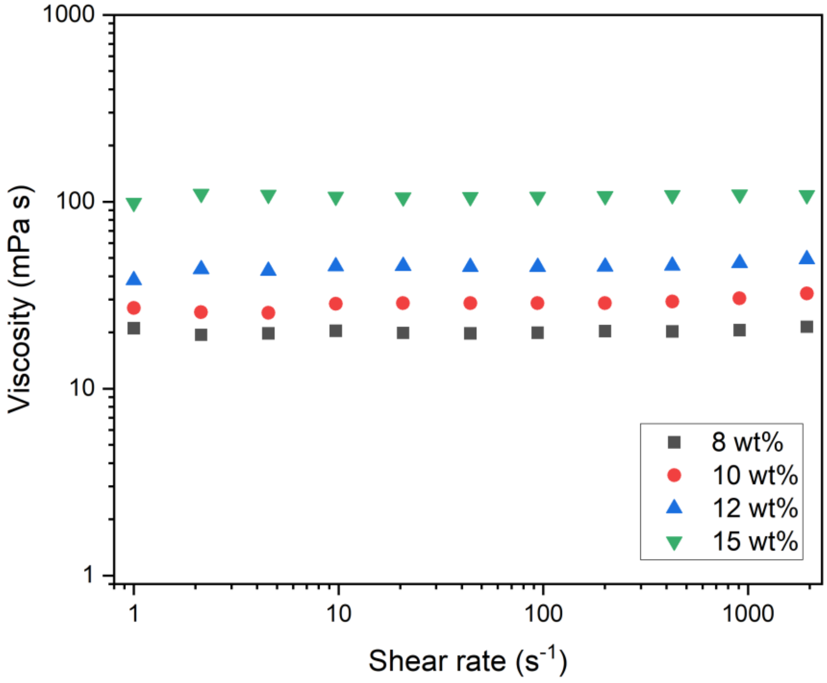

Figure S1. Viscosity vs shear rate for the inks having 8-15wt% of PVDF-TrFE 80/20 mol% at 25°C.

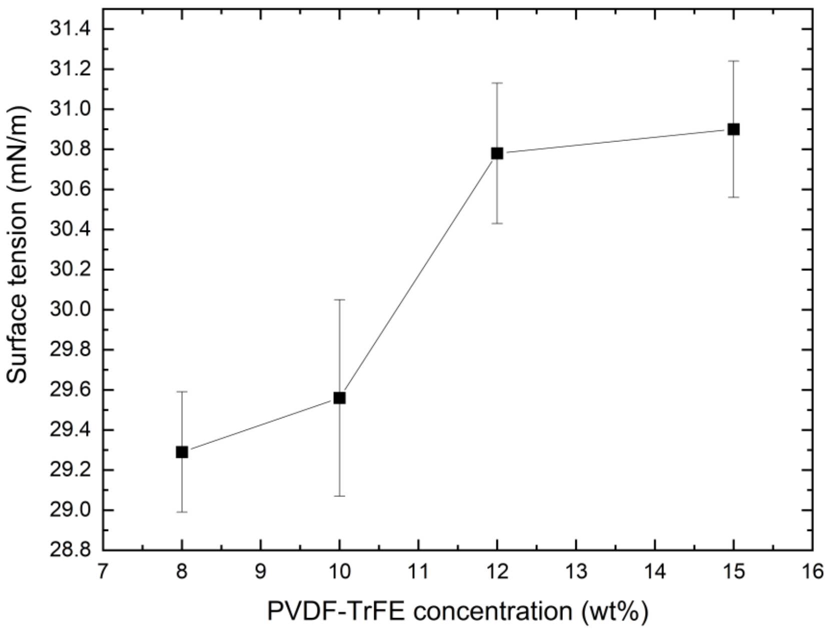

Figure S2. Surface tension measurements of solutions with different concentrations (wt%) of PVDF-TrFE 80/20 %mol in DMSO/Acetone mixture.

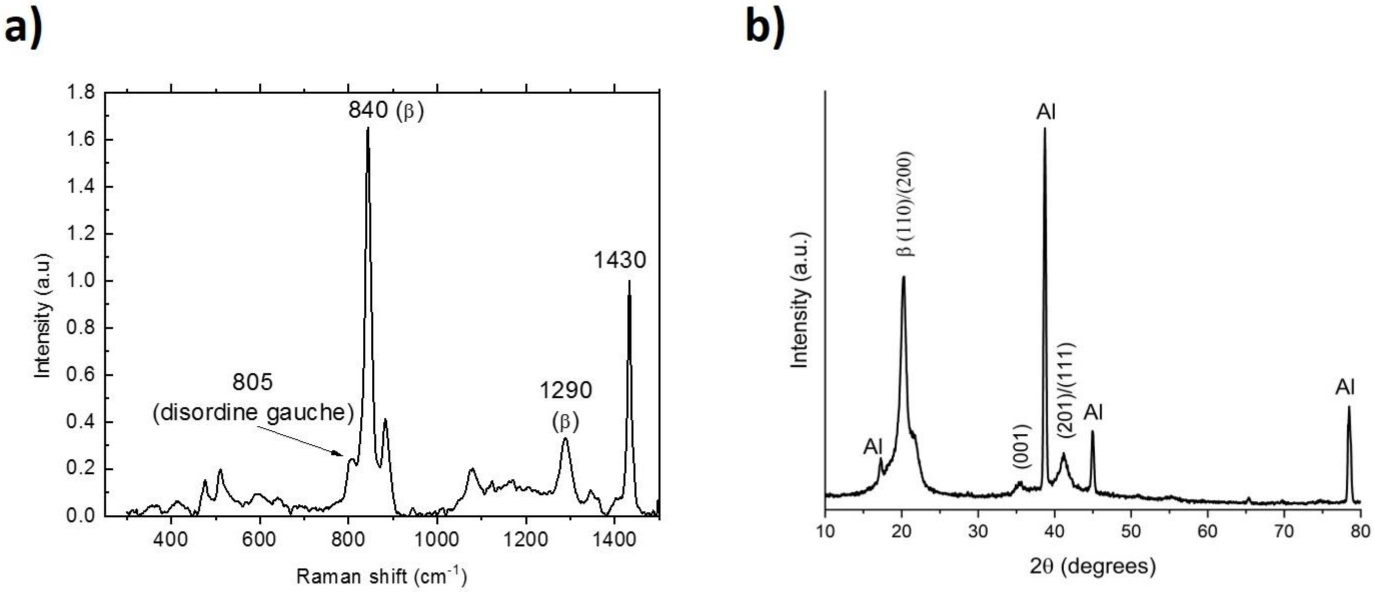

Figure S3. Raman (a) and XRD (b) spectra of a multilayer PVDF-TrFE 80/20 mol% film deposited by gravure printing and post-annealed at 120°C for 1h.

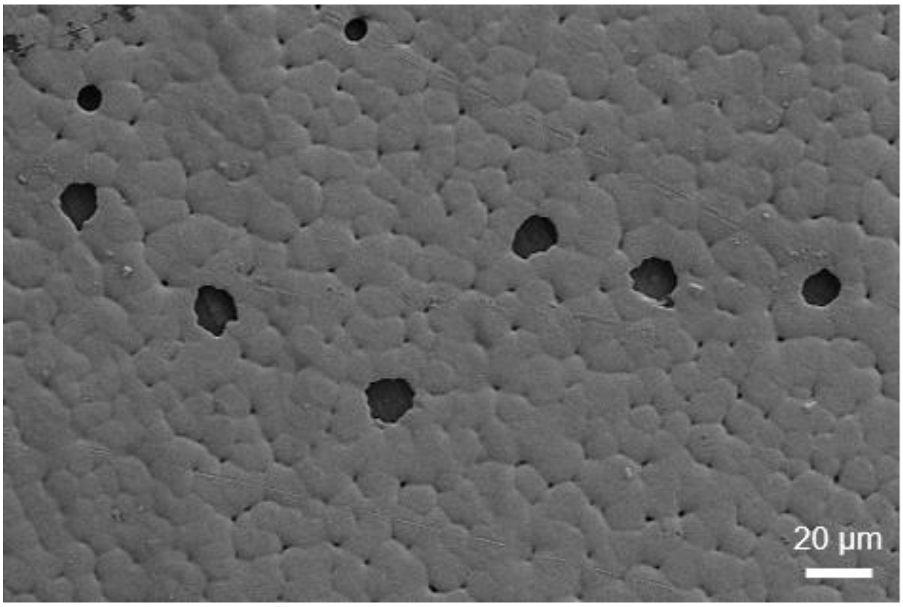

Figure S4. Top surface of gravure printed PVDF-TrFE film corona poled at 9.5 kV, 30°C for 1 min.

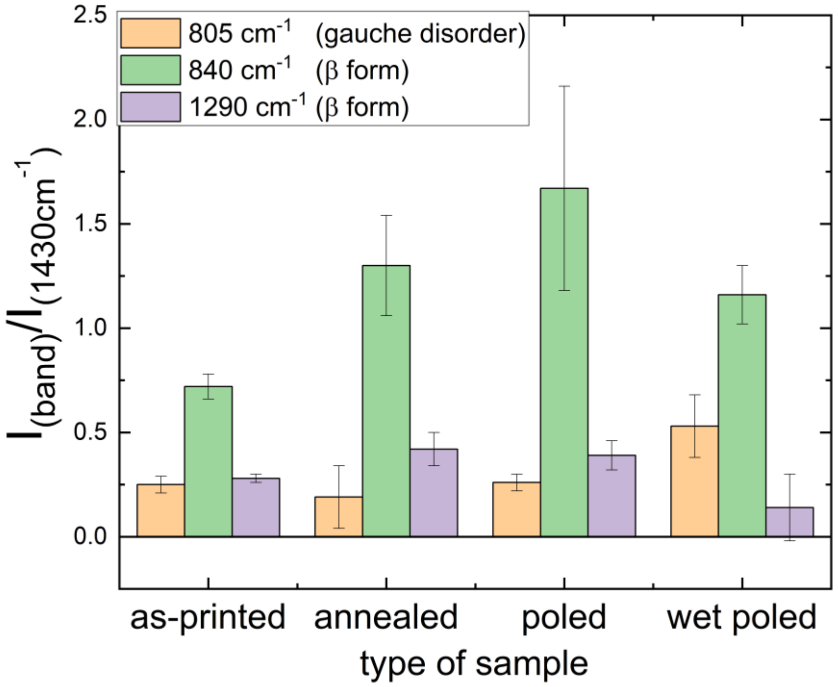

Figure S5. Comparison among the normalized intensities (respect to the band centered at 1430cm^{-1}) of Raman bands associated to different crystalline phases of PVDF-TrFE copolymers estimated for gravure printed samples: as-printed, thermal annealed at 120°C for 1 h, corona poled (at 9 kV, 30°C, 1 min) and wet poled (at 9 kV, 100°C, 30 min).

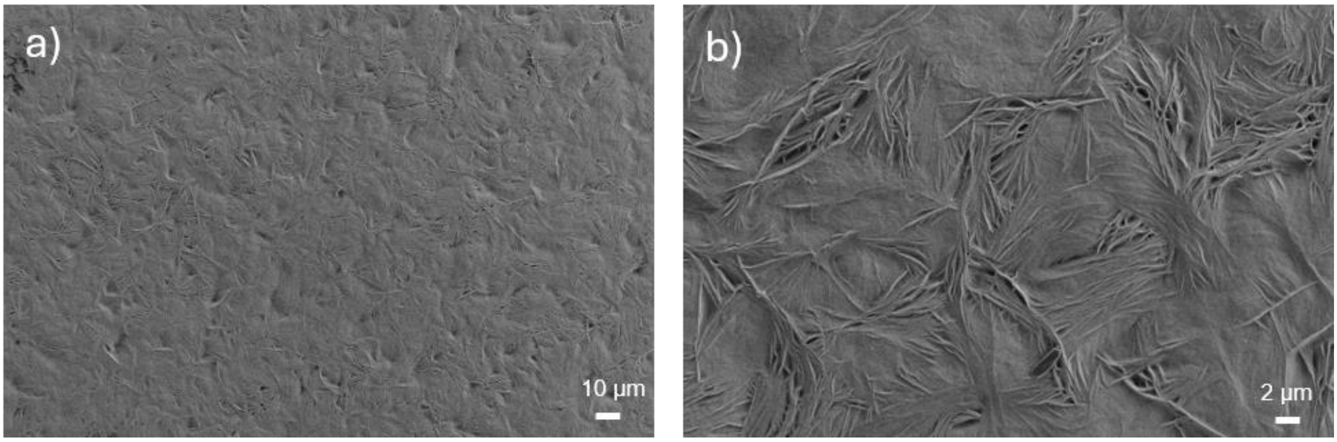

Figure S6. Top surface of gravure printed PVDF-TrFE film corona poled (at 9 kV, 30°C for 1 min) at different magnification: (a) 1 kX; (b) 5 kX.

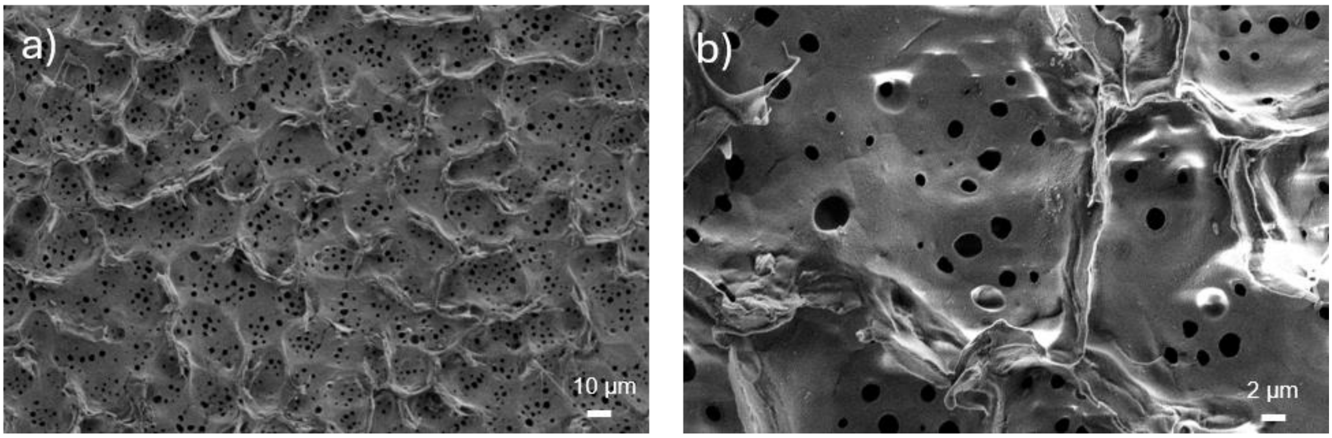

Figure S7. Top surface of wet poled (at 9 kV, 100°C isotherm for 30 min) of gravure printed PVDF-TrFE films at different magnification: (a) 1 kX; (b) 5 kX.
